# Immunodiversity of the *Arabidopsis* ZAR1 NLR Is Conveyed by Receptor-Like Cytoplasmic Kinase Sensors

**DOI:** 10.3389/fpls.2020.01290

**Published:** 2020-08-21

**Authors:** Alexandre Martel, Bradley Laflamme, Derek Seto, D. Patrick Bastedo, Marcus M. Dillon, Renan N. D. Almeida, David S. Guttman, Darrell Desveaux

**Affiliations:** ^1^ Department of Cell and Systems Biology, University of Toronto, Toronto, ON, Canada; ^2^ Centre for the Analysis of Genome Evolution & Function, University of Toronto, Toronto, ON, Canada

**Keywords:** effector-triggered immunity, immunodiversity, *Pseudomonas syringae*, receptor-like cytoplasmic kinase (RLCK), ZAR1, ZED/ZRK, *Arabidopsis*

## Abstract

The *Arabidopsis* nucleotide-binding leucine-rich repeat protein ZAR1 can recognize at least six distinct families of pathogenic effector proteins to mount an effector-triggered immune response. This remarkable immunodiversity appears to be conveyed by receptor-like cytoplasmic kinase (RLCK) complexes, which associate with ZAR1 to sense several effector-induced kinase perturbations. Here we show that the recently identified ZAR1-mediated immune responses against the HopX1, HopO1, and HopBA1 effector families of *Pseudomonas syringae* rely on an expanded diversity of RLCK sensors. We show that individual sensors can recognize distinct effector families, thereby contributing to the expanded surveillance potential of ZAR1 and supporting its role as a guardian of the plant kinome.

## Introduction

Many Gram-negative phytopathogenic bacteria subvert host immunity using a type III secretion system (T3SS), which translocates virulence-associated type III secreted effectors (T3SEs) directly into host cells (Dangl et al., 2013; Buttner, 2006; Khan et al., 2018). To guard against T3SEs, plant hosts have evolved mechanisms to detect T3SEs and mount a robust immune response, termed effector-triggered immunity (ETI) (Buttner, 2006; Dangl et al., 2013; Khan et al., 2016). ETI is mediated by plant nucleotide-binding, leucine-rich repeat (NLR) proteins, which either directly or indirectly detect the activities of specific T3SEs (Jones and Dangl, 2006; Khan et al., 2016). These ETI responses can be associated with a hypersensitive cell death response (HR), which is associated with greater reductions in pathogen growth (Laflamme et al., 2020).

A recent systematic investigation of the ETI landscape of the *Pseudomonas syringae* – *Arabidopsis thaliana* (hereafter *Arabidopsis*) model pathosystem revealed that ETI is a pervasive form of immunity, where nearly all *P. syringae* strains carry at least one T3SE capable of eliciting ETI (Laflamme et al., 2020). This near complete immunity against *P. syringae* can be conferred by a small number of NLR proteins. A prominent NLR against *P. syringae* is ZAR1, which can recognize five distinct T3SE families (HopZ1, HopX1, HopF1/HopF2, HopO1, and HopBA1) found in 199/494 (40.3%) of the strains studied (Laflamme et al., 2020). ZAR1 also mediates the recognition of AvrAC, a T3SE from *Xanthomonas campestris* (Wang et al., 2015). This remarkable immunodiversity is conferred by two families of ZAR1-associated receptor-like cytoplasmic kinases (RLCKs): ZED1-related kinases (ZRKs or RLCK family XII) and PBS1-like kinases (PBLs or RLCK family VII) (Lewis et al., 2013; Wang et al., 2015; Seto et al., 2017; Bastedo et al., 2019; Liu et al., 2019). This association between ZAR1 and RLCKs extends beyond *Arabidopsis*, since the RLCK family XII member JIM2 acts with *Nicotiana benthamiana* ZAR1 to recognize the T3SE XopJ from *Xanthomonas perforans* (Lewis et al., 2013; Schultink et al., 2019).

The requirement of RLCKs for *Arabidopsis* ZAR1-mediated immunity has been demonstrated for the recognition of the *X. campestris* T3SE AvrAC and the *P. syringae* T3SEs HopZ1a and HopF1r (formerly HopF2a) (Lewis et al., 2013; Wang et al., 2015; Seto et al., 2017). Uridylylation of PBL2 by AvrAC promotes its interaction with ZAR1-associated RKS1 (also known as ZRK1) (Wang et al., 2015). Cryo-electron microscopy structures of the PBL2-RKS1-ZAR1 complex indicate that uridylylated PBL2 promotes conformational changes of the ZAR1/RLCK complex resulting in ADP/ATP exchange and the formation of a pentameric resistosome structure, which triggers immunity (Wang et al., 2019a; Wang et al., 2019b). A similar mechanism has been proposed for the recognition of HopZ1a (Hu et al., 2020). HopZ1a acetylates ZED1 and modifies the interaction between ZED1 and multiple PBLs to activate ZAR1 (Lewis et al., 2010; Bastedo et al., 2019). The ZED1 autoimmune allele, ZED1-D, requires the two PBL kinases, SZE1 and SZE2 to mediate ZAR1-dependent autoimmunity (Liu et al., 2019). SZE1 and SZE2 also function redundantly in HopZ1a ETI (Liu et al., 2019). HopF1r is an ADP-ribosyltransferase that requires ZRK3 to trigger ZAR1 ETI (Seto et al., 2017). However, HopF1r does not ADP-ribosylate ZRK3 suggesting that another protein (e.g. PBL) is involved in the recognition of this T3SE. Overall, these examples suggest that ZAR1 uses RLCK complexes as sensors to detect a broad range of T3SE-induced kinase modifications and expand its surveillance potential.

Here, we provide further support for this model by characterizing the genetic requirements of the recently identified ZAR1-dependent ETI-eliciting T3SE families. We show that HopX1, HopO1, and HopBA1 ETI responses require ZED1, ZRK3, and ZRK2, respectively, providing the first evidence that individual ZED1/ZRK kinases can contribute to the recognition of multiple T3SE families. We further show that HopX1 promotes the interaction between ZED1 and the PBL kinase SZE1, and that SZE1 is required for HopX1-triggered immunity. Finally, we observe that the distribution of ZAR1-dependent ETI-eliciting alleles across *P. syringae* strains is largely non-redundant, where only five strains out of 150 carry multiple T3SEs that trigger ZAR1-mediated ETI. Our results suggest that ZAR1 uses combinations of ZRK/PBL complexes as sensors to recognize kinase perturbations induced by distinct T3SE families and provide broad spectrum immunity against *P. syringae*.

## Materials and Methods

### Plant Material

All *Arabidopsis* plants were grown under the following conditions in Sunshine Mix 1 soil: 12-h photoperiod, 150 microeinsteins of light, constant 22°C. 3- to 5-week-old plants were used for spray inoculation or hypersensitive response assays.

### Plant Mutant Lines

Information pertaining to all plant mutants used in this study are presented in **Supplementary Table 1**. *sze1-4* was generated through CRISPR/Cas9-mediated mutagenesis (Xing et al., 2014) using two guides RNAs (gRNA1: 5ʹ-TTGGGAGCATTTGGTTCGG-3ʹ; gRNA2: 5ʹ- TAAGTCTTCCCGCTTCAAG-3ʹ) that were designed using CHOPCHOP (Labun et al., 2019) and cloned into pBEE401E (Doucet et al., 2019). Basta-resistant T1 seeds that underwent heat treatment intervals to increase the number of positive knockouts (LeBlanc et al., 2018) were screened using the following primers (Forward 5ʹ-TCAGCCAGAAGAGAATTAGGG-3ʹ, Reverse 5ʹ-TGCGCCAAATTTCAAGACC-3ʹ). Sanger sequencing of the amplified PCR fragments confirmed that the *SZE1* locus had been disrupted (**Supplementary Figure 4**).

### Bacterial Infections

All *P. syringae* strains used in this study have been previously described (Laflamme et al., 2020). For spray inoculation, strains were grown overnight at 30°C on KB agar supplemented with rifampicin (50 μg/ml) and kanamycin (50 μg/ml). Bacteria were resuspended in 10 mM MgSO_4_ with 0.04% silwet L-77 and diluted to an OD_600_ = 0.8. 3- to 4-week-old plants were sprayed with approximately 2.5 ml of bacterial culture per plant using Preval sprayers and immediately domed. Disease symptoms developed over the next 10 days, during which the plants were photographed using a Nikon D5200 DSLR camera affixed with a Nikon 18 to 140 mm DX VR lens.

Bacterial growth assays were performed 3 days post infection. Four leaf discs (total leaf surface area = 1 cm^2^) were harvested from each plant. Leaf discs were homogenized in 1 ml of 10 mM MgSO_4_ using a bead-beater from which serial dilutions were performed. 5 μl aliquots from each dilution was plated onto KB agar with rifampicin (50 μg/ml) and were grown overnight at 30°C, at which point colonies were counted.

### Hypersensitive Response Assays

Hypersensitive response assays were performed on 4- to 5-week-old plants. *P. syringae* strains of interest were grown overnight at 30°C on KB rifampicin (50 μg/ml) and kanamycin (50 μg/ml) then resuspended in 10 mM MgSO_4_ and diluted to an OD_600_ = 0.2. This diluted culture was syringe-infiltrated into the left side of each tested leaf. Tissue collapse was assessed 18 to 22 h post-infiltration. Leaves were imaged using a Nikon D5200 DSLR camera affixed with a Nikon 18 to 140 mm DX VR lens.

### Quantification of Protein-Protein Interactions

To assess protein-protein interactions, genes of interest were cloned into the pEG202/pJG4-5 set of yeast two-hybrid vectors. pEG202 (*HIS* ^+^) constructs were carried in the haploid yeast strain EGY48 (alpha mating type), while pJG4-5 (*TRP* ^+^) constructs were carried in the haploid yeast strain RFY206 (A mating type), which also carried the *lacZ* reporter plasmid pSH18-34 (*URA* ^+^). To assess protein-protein interactions in the presence of a third protein, we used the yeast three-hybrid system (Bastedo et al., 2019), where a third protein of interest is integrated at the chromosomal *HO* locus of the haploid yeast strain EGY48 that was obtained using the pBA2262 plasmid. Alternatively, the third protein was expressed from the autonomously-replicating, single copy plasmid pBA350V (*LEU* ^+^) (Lee et al., 2019). We show that both of these systems provide similar results (**Figure 3B**, **Supplementary Figure 5**).

EGY48 and RFY206 strains of interest were mated by co-incubating strains on non-selective YPD glucose agar for two days at 30°C. The yeast mixture then went through two rounds of selection (two days of growth at 30°C) on YNB glucose with the appropriate selection: -Ura -Trp -His (for strains carrying pEG202, pJG4-5, pSH18-34 and the *HO* locus integration) or -Ura -Trp -His -Leu (for strains carrying pEG202, pJG4-5, pSH18-34 and pBA350V). For qualitative assessment of protein-protein interactions, mated diploid yeast strains were plated onto YNB agar supplemented with 1% raffinose, 2% galactose, 0.05 M sodium phosphate, 10 mg/ml X-gal and the appropriate selection, grown at 30°C and monitored for 3 days.

Quantitative protein-protein interactions were performed using the “IV. D. liquid culture assay using PNPG as substrate” protocol in the “yeast protocols handbook” from Clontech Laboratories (Protocol No. PT3024-1, Version No. PR742227). Briefly, yeast strains were grown overnight at 30°C in YNB supplemented with 2% galactose and 1% raffinose and appropriate auxotrophic selection (YNB Gal/Raf). Overnight cultures were diluted in 5 ml of YNB Gal/Raf to an OD_600_ of 0.4 and allowed to grow for 3 to 4 h, or until the OD_600_ reached 0.5 to 0.8. Once cultures reached this stage, the OD_600_ of each sample was noted and 1.5 ml of culture was centrifuged at 14,000 rpm for 1 min, the supernatant was removed and the pellet was washed in 1× volume of Z buffer (60 mM Na_2_HPO_4_, 40 mM NaH_2_PO_4_, 10 mM KCl, 1 mM MgSO_4_, pH 7). Samples were pelleted and resuspended in 300 μl Z buffer (concentration factor = 5), then aliquoted into three new tubes, each containing 100 μl. Tubes were subjected to three liquid nitrogen freeze-thaw cycles (1 min in liquid nitrogen followed by 1 min in a 37°C water bath), after which 700 μl Z buffer (supplemented with beta-mercaptoethanol to 50 mM) was added. To start the reaction, 160 μl of 4 mg/ml ONPG (dissolved in Z buffer) was added to each tube. The reaction was allowed to progress for 20 to 45 min and was stopped with the addition of 400 μl of 1 M Na_2_CO_3_. Samples were centrifuged for 10 min at 14,000 rpm, then the OD_420_ was measured. Final reaction units were calculated using the following formula: U = (1000 × OD_420_)/(t × V × OD_600_), where U (units), OD_420_ (reading following reaction), t (duration of reaction), V (volume of culture in reaction tube × concentration factor; 0.1 ml × 5 in the described protocol) and OD_600_ (reading of culture prior to reaction).

Western blotting to confirm protein expression levels was performed by precipitation with trichloroacetic acid as previously described (Bastedo et al., 2019) using the same yeast culture that was used for quantitative protein-protein interaction analysis.

## Results

### Expanded ZAR1 Immunodiversity Is Conferred by ZED1-Related Kinases (ZRKs)

The HopX1, HopO1, and HopBA1 families of T3SEs were recently found to trigger ZAR1-dependent ETI in the Col-0 ecotype of *Arabidopsis* (Laflamme et al., 2020). We confirmed this requirement by spray inoculating five independent *zar1* knockout lines with *P. syringae* pv. *tomato* DC3000 (*Pto*DC3000) harboring an empty vector (EV) or an ETI-eliciting allele from the HopX1 (HopX1i), HopO1 (HopO1c), or HopBA1 (HopBA1a) T3SE families (**Supplementary Figure 1**). Compared to the ETI in wild-type plants, observed as healthy green plant tissue, ETI was lost in all five *zar1* lines for all three T3SEs, confirming that ZAR1 is required to mount ETI against the HopX1, HopO1, and HopBA1 T3SEs families (**Supplementary Figure 1**).

**Figure 1:**
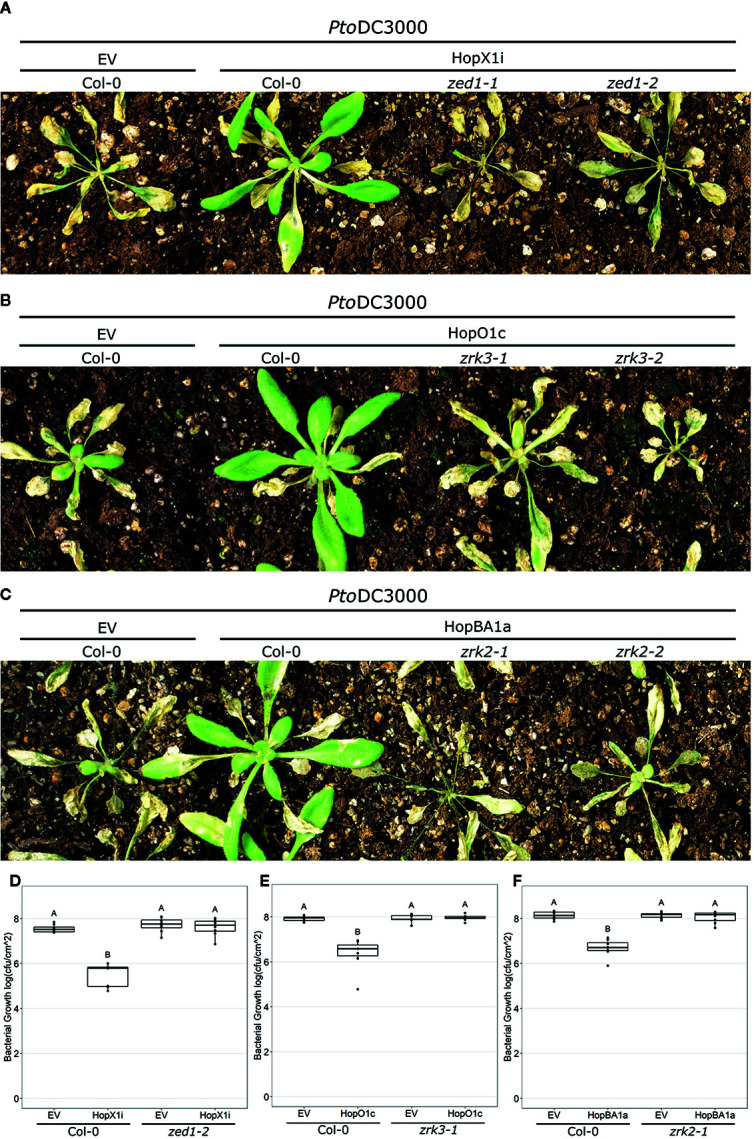
Expanded ZAR1 immunodiversity is conferred by ZED1-related kinases (ZRKs). **(A–C)** *Arabidopsis* Col-0 and two independent knockout lines of *zed1* **(A)**, *zrk3* **(B)**, and *zrk2* **(C)** were spray inoculated with *Pto*DC3000 carrying an empty vector (EV) or expressing HopX1i **(A)**, HopO1c **(B)**, or HopBA1a **(C)**. Images were taken 10 days post infection. **(D–F)** Bacterial growth assays 3 days post infection of *Pto*DC3000 EV or expressing HopX1i **(D)**, HopO1c **(E)**, or HopBA1a **(F)** on *Arabidopsis* Col-0 and *zed1-2* **(D)**, *zrk3-1* **(E)**, or *zrk2-1* **(F)**. Boxplots represent data from seven to eight samples. Letters represent statistically significant differences (Tukey’s HSD, P < 0.05). Experiments were replicated three times with similar results.

Since all previously characterized ZAR1-dependent ETI responses require ZRKs (Lewis et al., 2013; Wang et al., 2015; Seto et al., 2017), we investigated whether the HopX1, HopO1, and HopBA1 ETI responses were also ZRK-dependent. A reverse genetic screen for loss of HopX1i, HopO1c, or HopBA1a ETI in *zrk* knockout lines confirmed that all novel ZAR1-dependent immune responses are ZRK-dependent: HopX1i ETI requires ZED1, HopO1c ETI requires ZRK3, and HopBA1a ETI requires ZRK2 (**Figures 1A–C**; **Supplementary Figure 2**). These genetic requirements were confirmed using an additional independent *zrk* knock-out line for each ETI response (**Figures 1A–C**). Furthermore, the reduced *in planta* bacterial growth associated with HopX1i, HopO1c, and HopBA1a ETI responses in wild-type plants was lost in *zed1-2*, *zrk3-1*, and *zrk2-1* mutants, respectively (**Figures 1D–F**), confirming that the HopX1, HopO1, and HopBA1 ZAR1-dependent ETI responses require ZED1, ZRK3, and ZRK2, respectively.

The five ZAR1-dependent ETI-eliciting *P. syringae* T3SE families (HopZ1, HopX1, HopF1, HopO1, and HopBA1) differ in their capacity to trigger an HR in *Arabidopsis* Col-0 (Laflamme et al., 2020). We confirmed that HopZ1a and HopO1c ETI responses display HR-associated tissue collapse, whereas HopX1i, HopF1r, and HopBA1a do not under our experimental conditions (**Supplementary Figure 3A**). To further characterize the HopO1 HR phenotype, we confirmed that the putative ADP-ribosylation catalytic residues of HopO1c (E264D and E266D) were required for HopO1c HR (**Supplementary Figure 3B**; Laflamme et al., 2020). HopO1c HR also required ZAR1 and ZRK3 since HR-associated tissue collapse was lost in *zar1-1* and *zrk3-1* mutant plants (**Supplementary Figures 3C, D**). These results confirm that both virulence suppression and HR associated with HopO1c ETI require ZAR1 and ZRK3. More broadly, these results demonstrate that individual ZAR/ZRK combinations can trigger ETI with and without an associated HR (**Supplementary Figure 3A**).

### HopX1 ETI Requires the PBL Kinase SZE1 but Not SZE2

**Figure 2:**
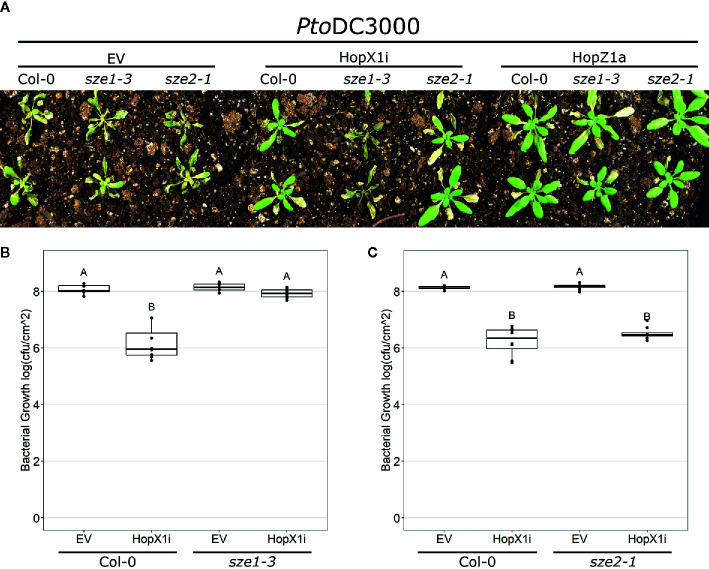
HopX1 ETI requires SZE1 but not SZE2. **(A)** *Arabidopsis* Col-0, *sze1-3*, and *sze2-1* were spray inoculated with *Pto*DC3000 EV, HopX1i, or HopZ1a. Images were taken 10 days post infection. **(B, C)** Bacterial growth assays 3 days post infection of *Pto*DC3000 EV or expressing HopX1i on *Arabidopsis* Col-0 and *sze1-3* **(B)**, or *sze2-1* **(C)**. Boxplots represent data from seven to eight samples. Letters represent statistically significant differences (Tukey’s HSD, P < 0.05). Experiments were replicated three times with similar results.

The PBL kinases SZE1 and SZE2 function redundantly in ZAR1 activation by HopZ1a and the auto-active ZED1 mutant *ZED1-D* (Liu et al., 2019). Since HopX1 ETI requires both ZAR1 and ZED1 (**Figure 1**, **Supplementary Figures 1** and **2**), we tested whether SZE1 or SZE2 are required for the recognition of HopX1. Spray inoculation of *Arabidopsis* Col-0 plants with *Pto*DC3000 harboring HopZ1a resulted in healthy green tissue associated with ETI in both the *sze1-3* and *sze2-1* knockout lines, while the green tissue associated with HopX1i ETI in *Arabidopsis* Col-0 plants was lost in *sze1-3* but not *sze2-1* plants (**Figure 2A**). We validated this phenotype with a second CRISPR knockout line (*sze1-4*), which also displayed a loss of ETI for HopX1i (**Supplementary Figure 4**). Loss of HopX1i ETI was confirmed by bacterial growth assays whereby *Pto*DC3000 harboring HopX1i grew to the same levels as *Pto*DC3000 EV on *sze1-3*, indicating that loss of SZE1 leads to complete abolishment of HopX1i ETI (**Figure 2B**). The bacterial growth reduction associated with HopX1 ETI was retained when assayed on *sze2-1* plants (**Figure 2C**). Thus, HopX1 ETI requires SZE1 but not SZE2.

### HopX1 ETI-Eliciting Alleles Promote ZED1-SZE1 Interaction

**Figure 3:**
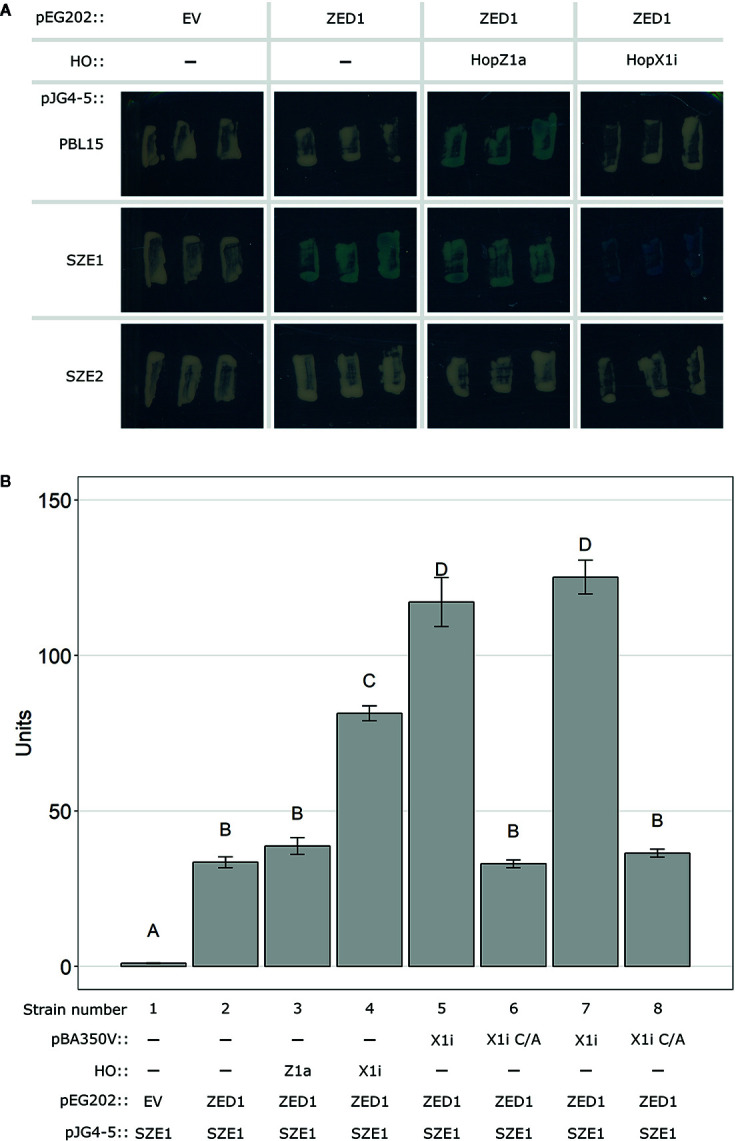
HopX1i strengthens the ZED1/SZE1 protein-protein interaction. **(A)** Yeast two-hybrid assay assessing interactions between ZED1 (in pEG202) and PBL15, SZE1, or SZE2 (in pJG4-5) on X-gal reporter plates. To determine protein-protein interaction in the presence of a T3SE, HopX1i, or HopZ1a was integrated into the yeast HO locus (see Methods). **(B)** Quantitative yeast interaction assays were performed between ZED1 and SZE1 in the presence or absence of the T3SEs HopZ1a (Z1a), HopX1i (X1i), or the catalytic mutant of HopX1i (C198A; X1i C/A). Letters represent statistically significant differences (Tukey’s HSD, P < 0.05). Experiments were replicated three times with similar results. Plasmids or genome integrations expressing each component for every strain used are listed below the bar graph. Strains 5 and 7 were independently generated, but harbor the same combinations of constructs (pBA350V::HopX1i, pEG202::ZED1, and pJG4-5::SZE1). Strains 6 and 8 were independently generated, but harbor the same combinations of constructs (pBA350V::HopX1i C182A, pEG202::ZED1, and pJG4-5::SZE1).

Dynamic interactions between ZRK and PBL kinases appear to play key roles in the activation of the ZAR1 NLR (Bastedo et al., 2019; Wang et al., 2019a; Wang et al., 2019b). As SZE1 and ZED1 are known to exist in a preformed complex (Liu et al., 2019), we assessed whether the presence of HopX1 can alter this interaction using a yeast three-hybrid system (Bastedo et al., 2019). We first investigated the ZED1 interaction dynamics with PBL15, SZE1, and SZE2 in the presence of HopZ1a or HopX1i. Consistent with previous *in planta* results, we observed an interaction between ZED1 and SZE1 (**Figure 3A**) (Liu et al., 2019). The presence of HopX1i increased the signal intensity of this interaction whereas HopZ1a did not (**Figure 3A**). This increase in signal intensity was specific to the SZE1/ZED1 interaction since HopX1i did not promote an interaction between ZED1 and SZE2, nor an interaction between PBL15 and ZED1, which is induced by HopZ1a (**Figure 3A**) (Bastedo et al., 2019). These results were confirmed using a liquid based quantification method (**Figure 3B**). Using this method, we also showed that promotion of the ZED1/SZE1 interaction by HopX1i was dependent on the catalytic residue C198, which is required for HopX1 ETI (**Figure 3B**) (Laflamme et al., 2020). Western blot analysis confirmed that the differences in yeast three-hybrid signal intensity were not due to differential protein accumulation (**Supplementary Figure 5A**). While HopX1 is proposed to act as a protease (Gimenez-Ibanez et al., 2014), we were unable to detect any cleavage products of either SZE1 or ZED1 when co-expressed with HopX1i (**Supplementary Figures 5B, C**).

To assess whether the increased interaction strength between ZED1 and SZE1 is specifically caused by ETI-eliciting HopX1 alleles, we repeated the quantitative yeast three-hybrid substituting HopX1i for a separate ETI-eliciting HopX1 allele, HopX1d, or a closely related allele that does not trigger ETI, HopX1b (Laflamme et al., 2020). Both ETI-eliciting HopX1 alleles (HopX1i and HopX1d) increased the ZED1-SZE1 interaction, whereas the presence of HopX1b (no ETI) did not (**Supplementary Figure 6**), suggesting that only ETI-eliciting HopX1 alleles are capable of increasing the ZED1-SZE1 interaction.

### Specific ZAR1-ZRK Modules Confer Resistance to Distinct *P. syringae* Clades

**Figure 4:**
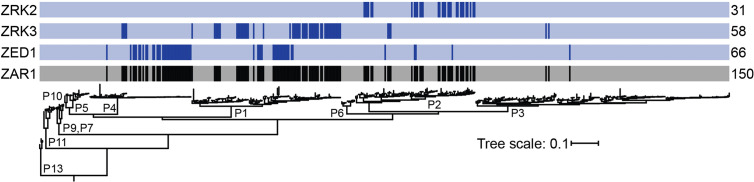
ZAR1 uses a diversity of kinase sensors to recognize *P. syringae*. Specific ZAR1/ZRK modules confer putative resistance to predominantly independent *P. syringae* clades. The generation of this *P. syringae* core genome phylogeny, with associated phylogroup designations (P) is described in Laflamme et al., 2020. Colored bars above the phylogeny represent strains that harbor an ETI eliciting allele that requires ZAR1 (black) and the specific ZRK required for each ZAR1 ETI (blue): ZED1, ZRK3 and ZRK2. Numbers indicate the total number of strains that carry a T3SE whose ETI requires the associated genetic component. Putatively truncated sequences (less than 75% the length of the representative allele) are not displayed (Laflamme et al., 2020).

ZAR1 has the capacity to recognize a significant number of diverse *P. syringae* strains (150/494; **Supplementary Table 2**) (Laflamme et al., 2020). To investigate the putative resistance spectrum that each ZAR1/ZRK module confers, we mapped the presence of ETI eliciting T3SE alleles from the HopZ1, HopX1, HopF1, HopO1 and HopBA1 families onto the core genome phylogeny of 494 P*. syringae* strains, as previously described (Laflamme et al., 2020), after removing alleles that were below 75% of the length of the screened T3SE. Subdividing T3SE recognition by ZAR1/ZRK module indicates that ZAR1/ZED1, ZAR1/ZRK2, and ZAR1/ZRK3 can potentially contribute to the recognition of 66, 31, and 58 strains, respectively (**Figure 4**, **Supplementary Table 2**). These strains are predominantly part of the primary *P. syringae* phylogroups 1, 2, and 4. Recognition of phylogroup 1 strains are split between the ZAR1/ZED1 and ZAR1/ZRK3 modules (**Figure 4**; 66/107). Phylogroup 2 strains are almost exclusively recognized by the ZAR1/ZRK2 module (**Figure 4**; 36/88), while the majority of phylogroup 4 strains are recognized by the ZAR1/ZED1 module (**Figure 4**; 45/75). Notably, there is virtually no overlap in the strains putatively recognized by all three ZAR1/ZRK modules and only five strains carry more than a single ZAR1 elicitor: *Pth*ICMP3934 harbors HopF1 and HopX1 (ZRK3 and ZED1), *Psy*USA007 harbors HopO1 and HopX1 (ZRK3 and ZED1), and *Psy*HS191, *Psy*CC457 and *Ps*A2 harbor HopBA1 and HopZ1 (ZRK2 and ZED1) (**Figure 4**, **Supplementary Table 2**). Therefore, the recognition spectrum of individual ZAR1/ZRK modules is associated with specific *P. syringae* phylogroups and these putative ETI responses are largely non-redundant.

## Discussion

**Figure 5:**
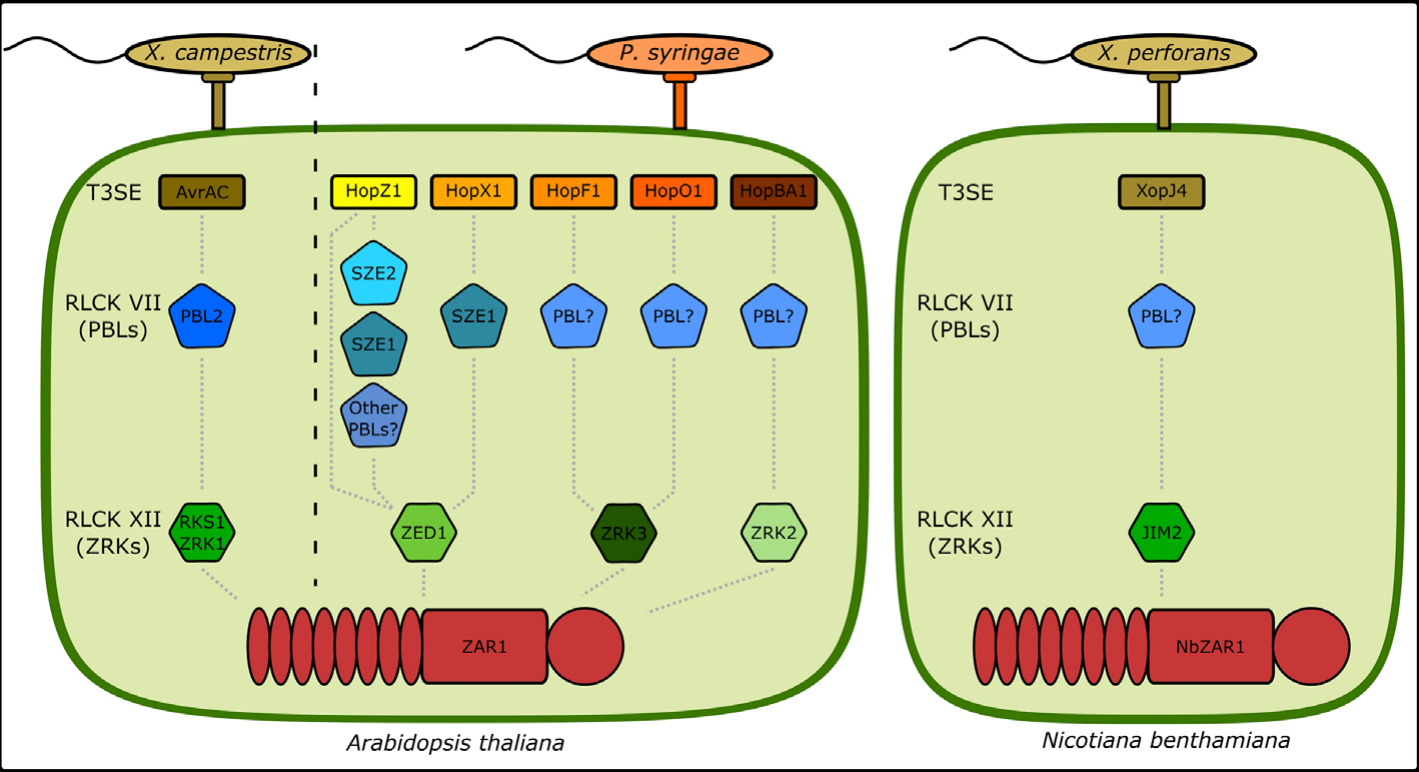
ZAR1 uses independent combinations of RLCKs to sense T3SE activity. A summary of all known genetic components involved in ZAR1-mediated ETI in *A. thaliana* and *N. benthamiana* to T3SEs from *P. syringae*, *X. campestris*, and *X. perforans*. *X. campestris* T3SE AvrAC uridylylation of PBL2 alters the conformation of the PBL2/RKS1/ZAR1 complex, resulting in ADP/ATP exchange in ZAR1 and the formation of a resistosome structure (Wang et al., 2019a; Wang et al., 2019b). HopZ1 activity modulates PBL/ZED1 interactions and its ETI was shown to redundantly require SZE1 and SZE2 (Bastedo et al., 2019; Liu et al., 2019). HopX1 ETI requires SZE1 and ZED1 (this study). HopF1 and HopO1 ETIs require ZRK3, but associated PBLs have not been identified (Seto et al., 2017) (this study). HopBA1 ETI requires ZRK2, but no associated PBLs have been identified (this study). Note that in the *Arabidopsis* Ag-0 ecotype, HopBA1 elicits ETI *via* the TIR-only NLR RBA1 (Nishimura et al., 2017). The *X. perforans* T3SE XopJ4 requires JIM2, an RLCK XII homologous to ZRKs, and NbZAR1 for ETI (Schultink et al., 2019).

We have shown that the ZAR1-mediated ETI responses against the *P. syringae* T3SEs HopX1, HopO1, and HopBA1 require ZED1, ZRK3, and ZRK2, respectively. This not only expands the recognition profile of the ZAR1/ZRK resistance modules, but also demonstrates that specific ZAR1/ZRK modules can recognize multiple T3SE families (**Figure 5**). For example, ZAR1/ZED1 recognizes HopZ1a and HopX1i, whereas ZAR1/ZRK3 recognizes HopF1r and HopO1c. Both HopF1r and HopO1c are ADP-ribosyltransferases which may similarly activate ZAR1/ZRK3, however HopZ1a and HopX1i appear to possess distinct enzymatic functions which may indicate that these complexes can recognize distinct kinase modifications (Lee et al., 2012; Gimenez-Ibanez et al., 2014). HopBA1 is the first effector identified to require ZRK2 for perception, however we speculate that additional ETI responses requiring members of the ZRK genomic cluster will be identified in future studies of other phytopathogens and their effectors (Lewis et al., 2010).

In addition to ZED1, HopX1 recognition requires the PBL kinase SZE1. Like AvrAC and the ZRK1/PBL2 complex, HopX1i can promote the interaction between ZED1 and SZE1 which may provide a similar mechanism for ZAR1 resistosome activation. However, promotion of ZED1/SZE1 interaction appears to be at odds with the proposed protease function of the HopX1 family. It is possible that the HopX1 protease function would produce fragments of either ZED1 or SZE1 that have higher affinity for the cognate partner, however we were unable to detect any such cleavage products (**Supplementary Figures 5B, C**). Alternatively, the HopX1 family may have biochemically diversified such that HopX1i possesses a distinct biochemical function from HopX1a, which was characterized as a protease (Gimenez-Ibanez et al., 2014). Since HopX1 possesses a catalytic triad that is characteristic of several enzymatic activities, it is also possible that these catalytic residues carry out additional enzymatic reactions, such as acetylation like HopZ1a which also activates ZAR1/ZED1. Overall, these results support the hypothesis that ZAR1 uses ZRK/PBL sensors to detect a diversity of kinase-targeted T3SE activities.

The use of kinase sensors may distinguish ZAR1 from most other NLRs which appear to function as pairs or networks of “sensor” NLRs that mediate recognition and “helper” NLRs that activate downstream immune signaling (Adachi et al., 2019). ZAR1 may represent a singleton NLR that functions as a “helper” NLR and associates with a myriad of kinase “sensors” to mediate effector recognition. Two additional NLRs that monitor for effector-induced perturbations of host kinases are RPS5 and Prf (Pedley and Martin, 2003; Shao et al., 2003; Ade et al., 2007). Although RPS5 ETI has only been associated with the kinase PBS1, Prf associates with multiple Pto-like kinases which can trigger ETI responses (Ntoukakis et al., 2009; Gutierrez et al., 2010). Tomato Prf is genomically located within a cluster of Pto-like kinases that are reminiscent of the ZRK cluster in *Arabidopis* (Salmeron et al., 1996; Lewis et al., 2013). Similar to ZAR1, Prf uses an expanded repertoire of associated kinases to diversify its effector recognition profile (Gutierrez et al., 2010). We speculate that effector-specificity in these NLR-kinase systems will be conferred by the differential ability of effectors to interact with and modify ZAR1-associated RLCKs to induce the structural rearrangements that promote resistosome formation.

The large number of kinase sensors monitored by ZAR1 would suggest that it should display high sequence conservation to retain its ability to interact with different kinase combinations and activate immunity. In contrast, these kinases, which are directly responsible for sensing T3SEs that are under heavy evolutionary pressures to overcome this recognition, would display less sequence conservation. In support of this, ZAR1 is highly conserved in the plant kingdom and displays a low non-synonymous mutation rate that is suggestive of purifying selection (Peele et al., 2014). In addition, a GWAS analysis for resistance to *X. campestris* in *Arabidopsis* uncovered RKS1 (ZRK1), which displayed signatures of balancing selection, suggesting that variation in ZAR1 resistance in *Arabidopsis* can be contributed to variation in ZRKs rather than in ZAR1 (Huard-Chauveau et al., 2013). Compared to other well-characterized *Arabidopsis* NLRs such as RPM1 and RPS5, whose presence/absence is known to vary across ecotypes, ZAR1 is conserved in all of the 1135 ecotypes included in the 1001 genomes project (Stahl et al., 1999; Tian et al., 2002; Consortium, 2016; Laflamme et al., 2020). ZAR1 orthologs have been identified in a multitude of plant species, including many outside of the Brassicaceae family (Peele et al., 2014; Schultink et al., 2019). *Nicotiana benthamiana* ZAR1 requires the kinase JIM2, which is closely related to ZED1, for recognition of XopJ, suggesting that kinase sensors are used by ZAR1 members outside the Brassicaceae family (Schultink et al., 2019).

Interestingly, ZAR1 ETI can occur with (HR+) or without (HR-) a macroscopic HR (Laflamme et al., 2020). In addition, we have shown that a single ZAR1/ZRK combination can produce HR+ and HR- ETI (**Supplementary Figure 3**). For example, HopZ1a (HR+) and HopX1i (HR-) both require ZAR1 and ZED1 for recognition but differ in their HR promoting ability. A possible explanation for this is that the presence or absence of an HR reflects the “amplitude” of ZAR1/ZRK activation. HopZ1a appears to promote the interaction of several PBL kinases with ZED1 and both SZE1 and SZE2 contribute to HopZ1a ETI (Bastedo et al., 2019; Liu et al., 2019). On the other hand, HopX1i promotes a more specific interaction between SZE1 and ZED1, and HopX1i ETI only requires SZE1 (**Figure 3**). It is possible that T3SEs that activate multiple kinase sensors promote HR+ ETI (e.g. HopZ1a) whereas those that activate a single sensor (eg. HopX1i) promote HR- ETI. It is also possible that certain kinase sensors are more prone to promote HR+ ETI than others.

These differences in HR elicitation by T3SEs that converge onto the same ZAR1-associated kinase raise the question of how an NLR can mediate these distinct immune outputs? The formation of a funnel-shaped structure by the N-terminal helices of the ZAR1 resistosome suggests that this structure may directly form a pore in the plasma membrane, resulting in cell death (Wang et a1., 2019a). Since the only two T3SEs demonstrated to promote ZAR1 resistosome formation also elicit HR (AvrAC and HopZ1a), it is possible that non-HR eliciting T3SEs result in an alternative ZAR1 structure, thereby explaining the difference in HR phenotypes (Wang et al., 2019a; Wang et al., 2019b; Hu et al., 2020). Conversely, it is possible that the different T3SEs that are recognized by ZAR1 all activate a resistosome structure, but do so with different efficiencies, thereby resulting in the observed differences in ZAR1-mediated immune outputs. These may include ETI-responses that completely lack HR or manifest a microscopic HR response that is not observed macroscopically. Finally, it is possible that additional components specifically mediate HR, such as the TIR-only protein RBA1, which mediates HR to HopBA1 in the Ag-0 ecotype of *Arabidopsis* (Nishimura et al., 2017).

In conclusion, ZAR1 uses a diversity of kinase combinations to sense the activity of multiple independent T3SE families from different phytopathogenic species. This variety of kinase sensors putatively allows ZAR1 to confer significant, and largely non-redundant, protection against diverse *P. syringae* strains (**Figure 4**). The modular nature of ZAR1 recognition makes it a candidate for tailored genetic engineering, where a desired resistance spectrum may be obtained by using specific kinase combinations.

## Data Availability Statement

The raw data supporting the conclusions of this article will be made available by the authors, without undue reservation.

## Author Contributions

AM, BL, DG, and DD designed experiments. AM and BL, performed experiments. AM, BL, DS, DB, MD, RA, DG, and DD generated tools and analyzed data. AM, DG, and DD wrote the manuscript.

## Funding

This project was supported by Natural Sciences and Engineering Research Council of Canada (NSERC) postgraduate awards (AM, BL, and DS), NSERC Discovery grants (DG and DD), a Canada Research Chair in Plant-Microbe Systems Biology (DD), and the Centre for the Analysis of Genome Evolution and Function (DD and DG).

## Conflict of Interest

The authors declare that the research was conducted in the absence of any commercial or financial relationships that could be construed as a potential conflict of interest.
