# Supplementary material for: Immunodiversity of the Arabidopsis ZAR1 NLR Is Conveyed by Receptor-Like Cytoplasmic Kinase Sensors
Source: Front Plant Sci. 2020 Aug 21;11:1290. doi: 10.3389/fpls.2020.01290 (PMC7475706; doi:10.3389/fpls.2020.01290)
Supplement: Supplementary file 1 [file Presentation_1.pdf]

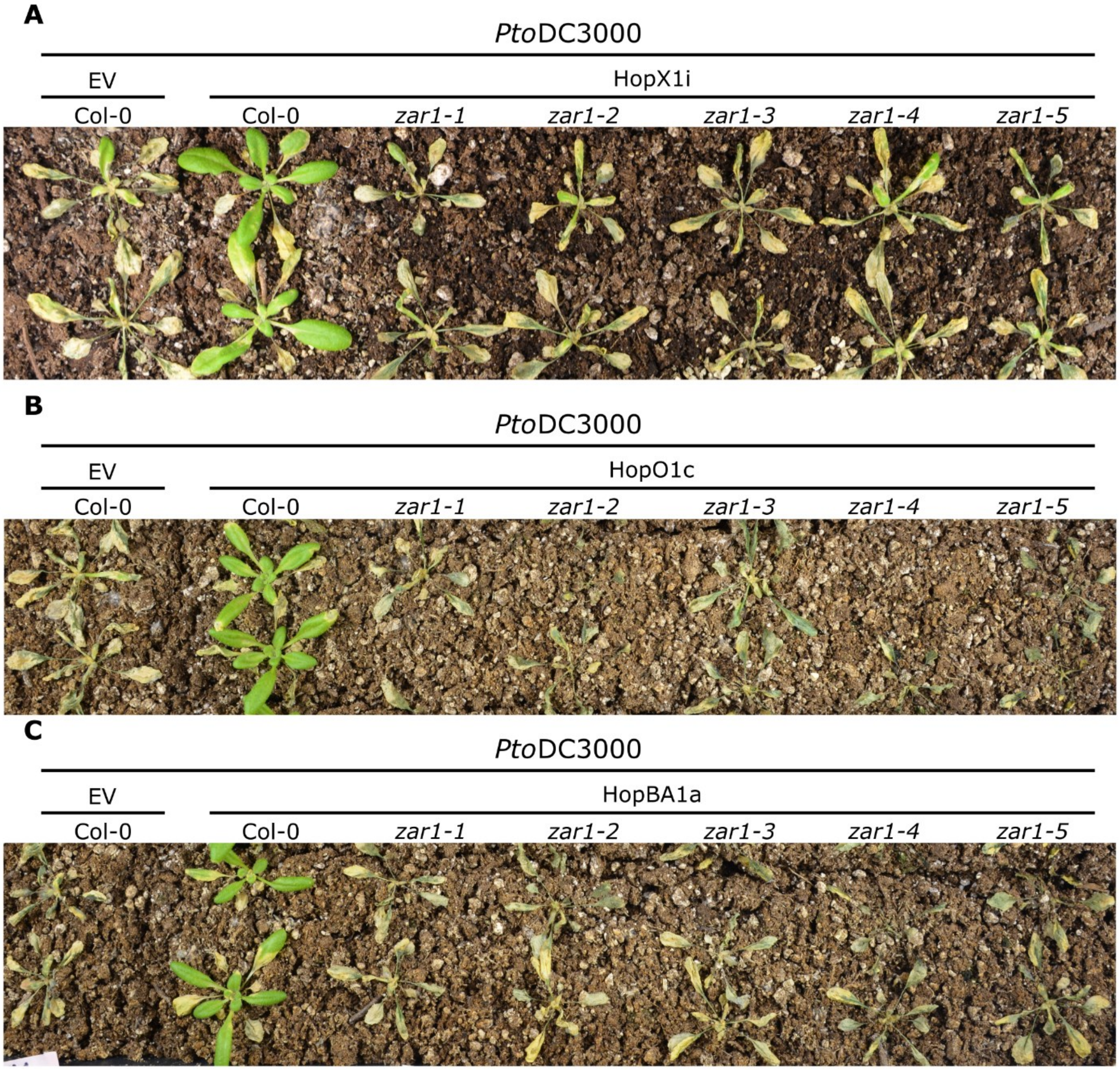

Supplementary Figure 1 Confirmation of ZAR1 dependence for HopX1, HopO1, and HopBA1 ETIs. Arabidopsis Col-0 and five independent ZAR1 knockout lines were spray inoculated with *Pto*DC3000 EV or expressing HopX1i **(A)**, HopO1c **(B)** or HopBA1a **(C)**. Images were taken 10 days post infection. Experiments were replicated three times with similar results.

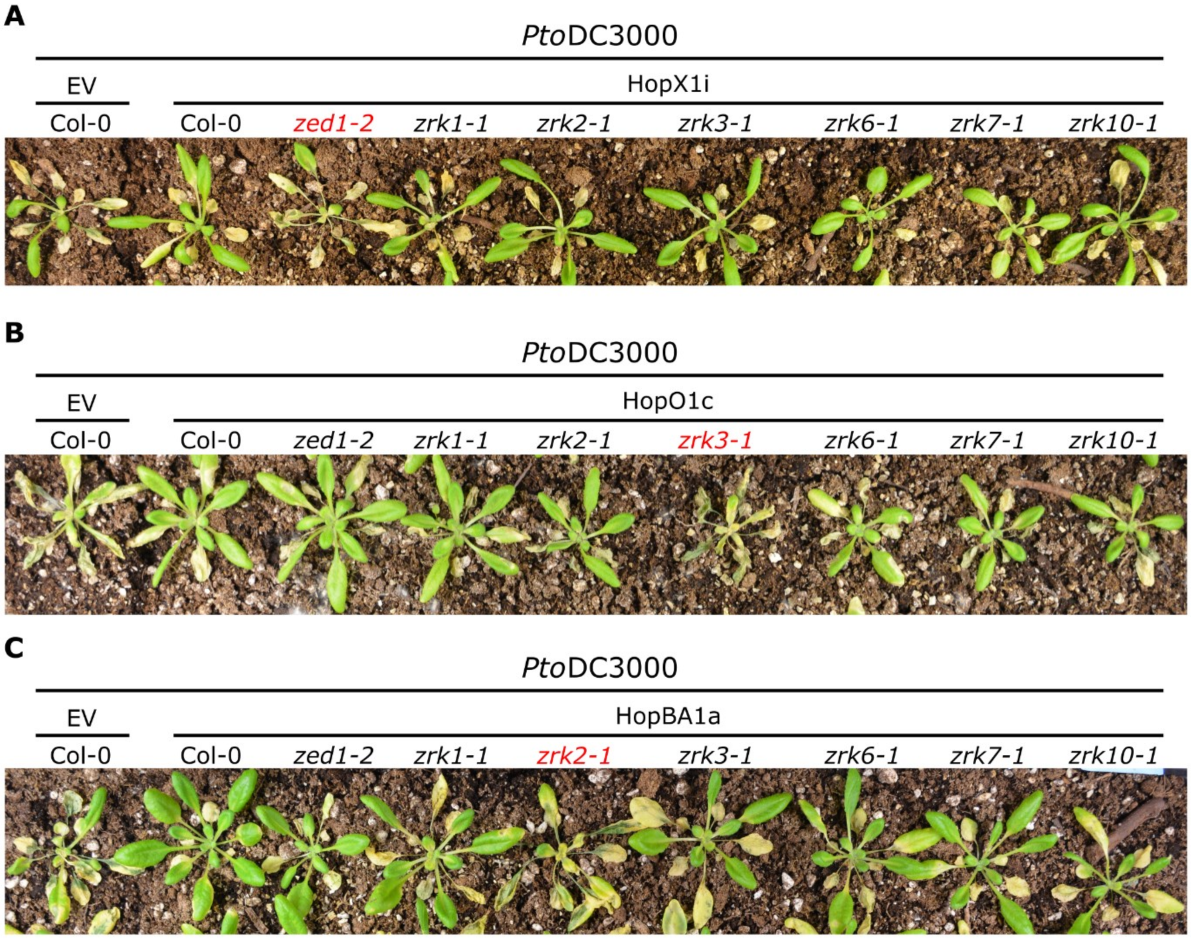

Supplementary Figure 2 Screening of ZAR1-dependent ETI responses on ZRK knockout lines. Arabidopsis Col-0 and knockout lines for ZRKs present within the ZRK genomic cluster (Lewis et al., 2010) were spray inoculated with *Pto*DC3000 EV or expressing HopX1i **(A)**, HopO1c **(B)**, or HopBA1a **(C)**. Images were taken 7-10 days post infection. Knockout lines which displayed a loss of ETI for an associated ETI elicitor are listed in red and were reconfirmed with an independent T-DNA insertion line (see Figure 1). Lines which did not display a loss of ETI were not reconfirmed with an independent line. Experiments were replicated three times with similar results.

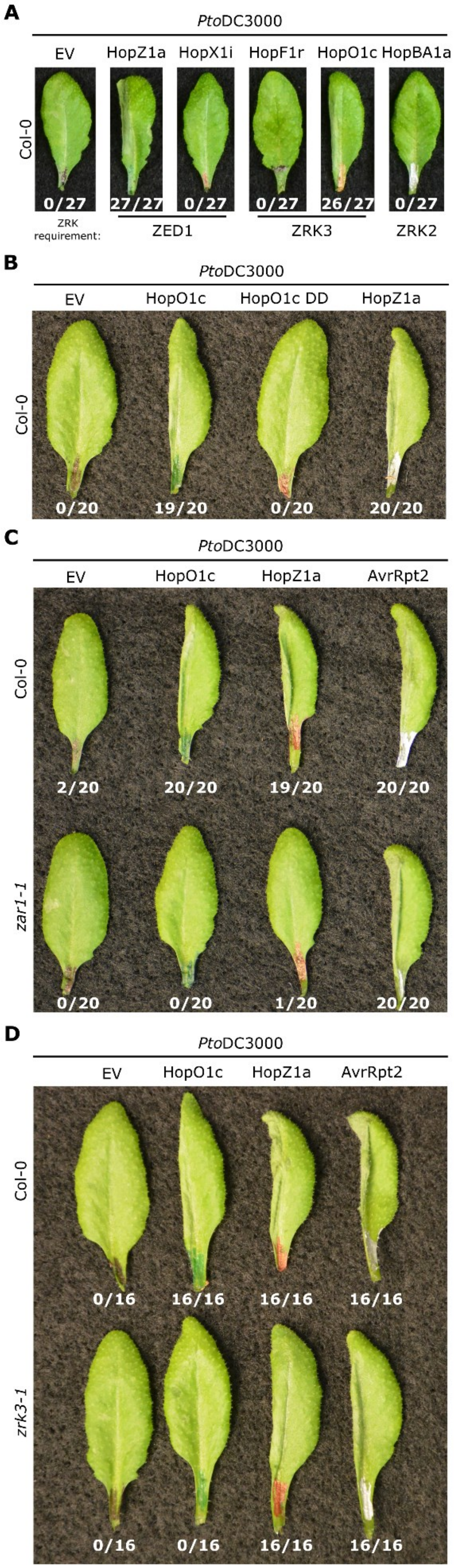
A
PtoDC3000
EV
HopZ1a
HopX1i
HopF1r
HopO1c
HopBA1a
Col-0
0/27
27/27
0/27
0/27
26/27
0/27
ZRK requirement:
ZED1
ZRK3
ZRK2
B
PtoDC3000
EV
HopO1c
HopO1c DD
HopZ1a
Col-0
0/20
19/20
0/20
20/20
C
PtoDC3000
EV
HopO1c
HopZ1a
AvrRpt2
Col-0
2/20
20/20
19/20
20/20
zar1-1
0/20
0/20
1/20
20/20
D
PtoDC3000
EV
HopO1c
HopZ1a
AvrRpt2
Col-0
0/16
16/16
16/16
16/16
zrk3-1
0/16
0/16
16/16
16/16

Supplementary Figure 3 ZAR1-dependent ETIs differ in their HR profiles and HopO1c HR requires putative catalytic residues, as well as ZAR1 and ZRK3. (A) HR assays using *Pto*DC3000 EV, HopZ1a, HopX1i, HopF1r, HopO1c, and HopBA1a were performed on Arabidopsis Col-0. The associated ZRK requirement for each ETI is provided. **(B)** HR assays using *Pto*DC3000 EV, HopO1c, HopO1c DD (E264D and E266D, catalytic mutant) or HopZ1a were performed on Col-0. **(C-D)** HR assays using *Pto*DC3000 EV, HopO1c, HopZ1a, or AvrRpt2 were performed on Col-0 and *zar1-1* **(C)**, or *zrk3-1* **(D)**. For all HR assays, leaves were imaged 20 hours post infiltration. The proportion of leaves displaying macroscopic HR is presented below each representative leaf. Infiltrations were performed on the left side of each leaf. Experiments were replicated three times with similar results.

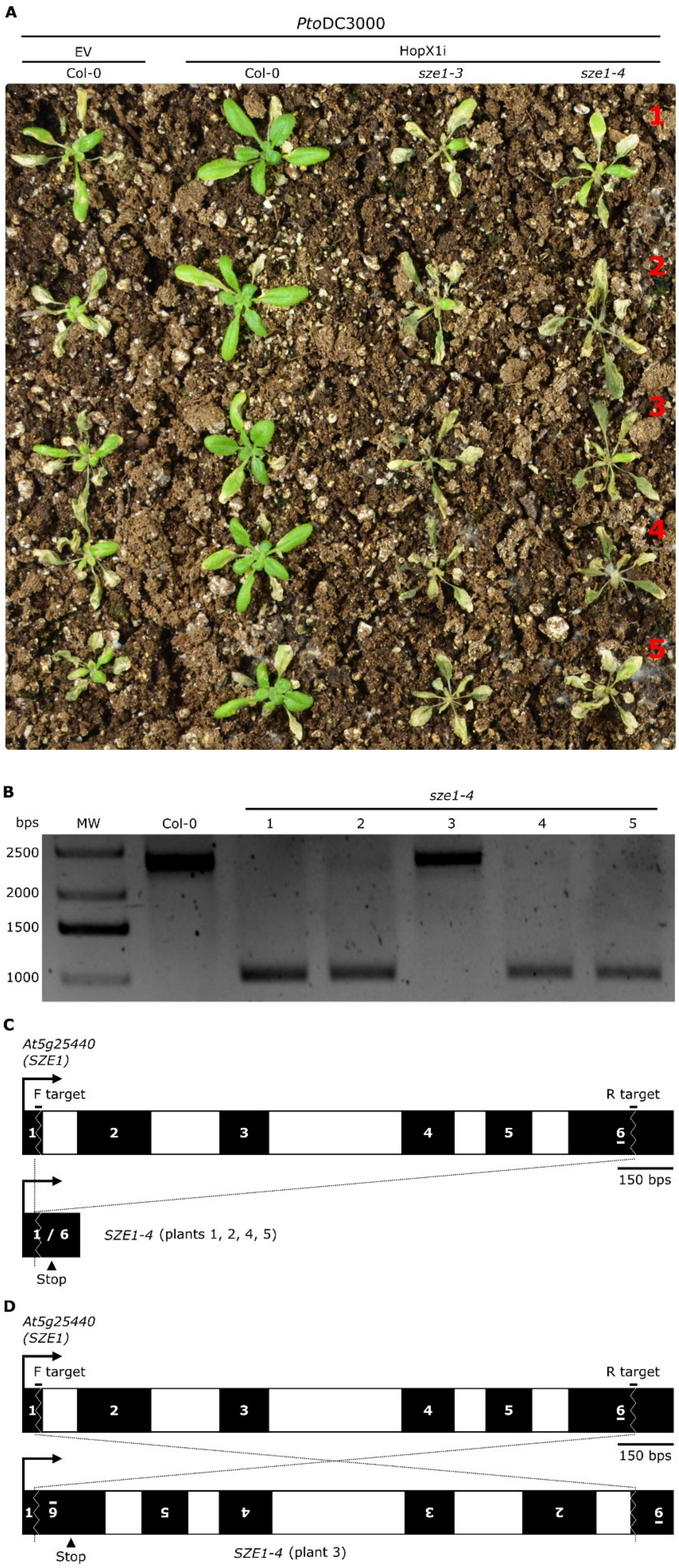
A
PtoDC3000
EV
HopX1i
Col-0
Col-0
sze1-3
sze1-4
1
2
3
4
5
B
sze1-4
bps
MW
Col-0
1
2
3
4
5
2500
2000
1500
1000
C
At5g25440
(SZE1)
F target
R target
1
2
3
4
5
6
150 bps
1 / 6
SZE1-4 (plants 1, 2, 4, 5)
Stop
D
At5g25440
(SZE1)
F target
R target
1
2
3
4
5
6
150 bps
SZE1-4 (plant 3)
Stop

Supplementary Figure 4 CRISPR-mediated knockout of SZE1 confirms its requirement for HopX1 ETI. (A) Arabidopsis Col-0, *sze1-3*, and *sze1-4* (T2 CRISPR-mediated knockout individuals) were spray inoculated with *Pto*DC3000 EV or expressing HopX1i. Images were taken 10 days post infection. **(B)** PCR screening of the *SZE1* locus in Col-0 and the five *sze1-4* individuals infected in **(A)**. The expected size for the deletion of interest is 1000 bps. MW: molecular weight marker. The PCR amplicon from each individual was sequenced to confirm that the *SZE1* locus was knocked out. **(C-D)** Visual representation of the sequencing results of the *SZE1* locus from *sze1-4* individuals 1, 2, 4 and 5 **(C)**, and individual 3 **(D)**. Regions filled in black represent exons, while white regions represent introns. “F target” and “R target” indicate the CRISPR guide target sites used. “Stop” indicates the presence of a stop codon.

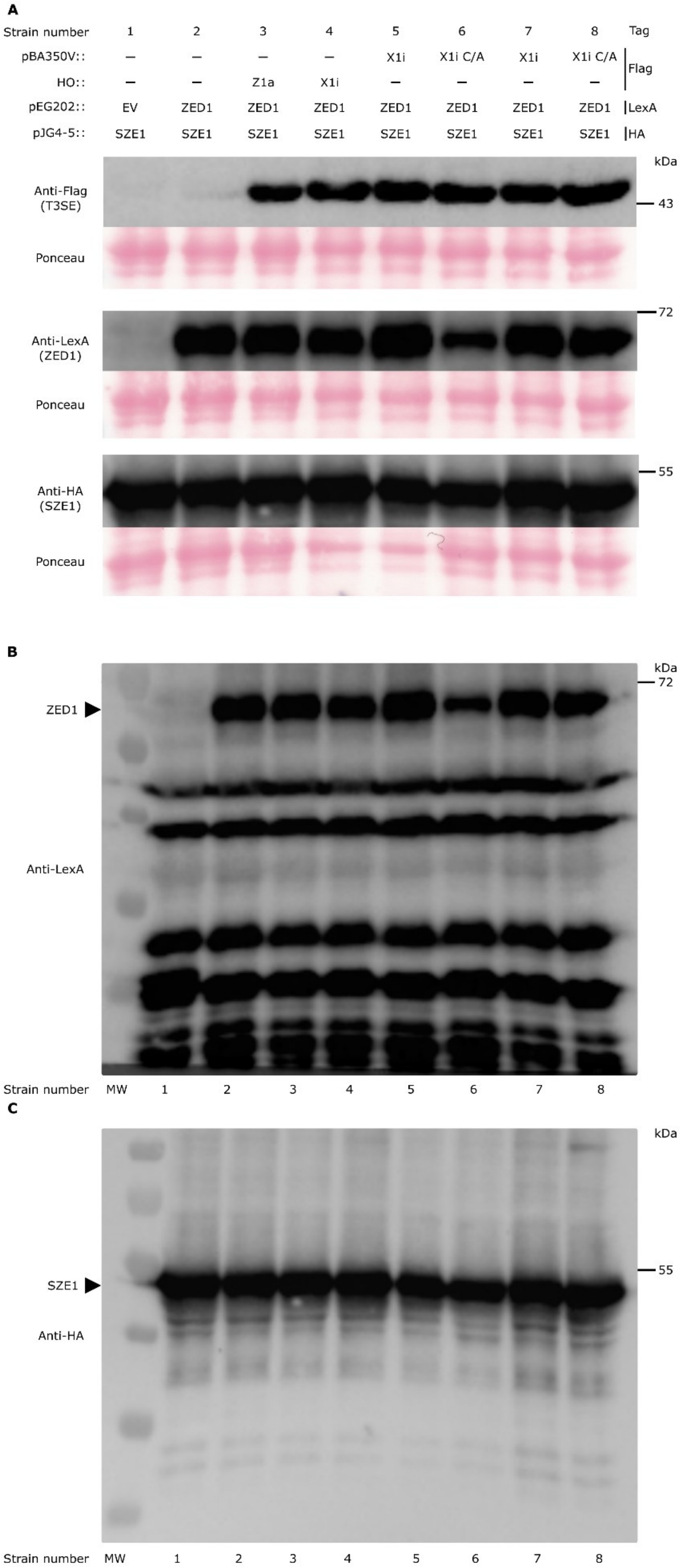
A
Strain number 1 2 3 4 5 6 7 8 Tag
pBA350V:: – – – – X1i X1i C/A X1i X1i C/A
HO:: – – Z1a X1i – – – –
Flag
pEG202:: EV ZED1 ZED1 ZED1 ZED1 ZED1 ZED1 ZED1 LexA
pJG4-5:: SZE1 SZE1 SZE1 SZE1 SZE1 SZE1 SZE1 SZE1 HA
kDa
Anti-Flag (T3SE)
43
Ponceau
72
Anti-LexA (ZED1)
Ponceau
55
Anti-HA (SZE1)
Ponceau
B
kDa
72
ZED1
Anti-LexA
Strain number MW 1 2 3 4 5 6 7 8
C
kDa
55
SZE1
Anti-HA
Strain number MW 1 2 3 4 5 6 7 8

Supplementary Figure 5 HopX1i strengthens ZED1-SZE1 protein-protein interaction in a catalytically dependent manner. Strain numbers refer to those presented in Fig. 3. Plasmids or genome integrations expressing each component for every strain used are listed below the strain number. The tag conferred by each vector/integration is listed on the right. Similar protein accumulation levels for all components co-expressed within the same yeast strain was confirmed by western blotting. **(B-C)** Expanded view of the LexA-tagged ZED1 **(B)** and HA-tagged SZE1 **(C)** western blots displayed in **(A)** which indicate a lack of observable cleavage product. The arrow indicates the bands that represent ZED1 **(B)** and SZE1 **(C)**. Strain numbers match the descriptions provided in **(A)**; MW = molecular weight marker.

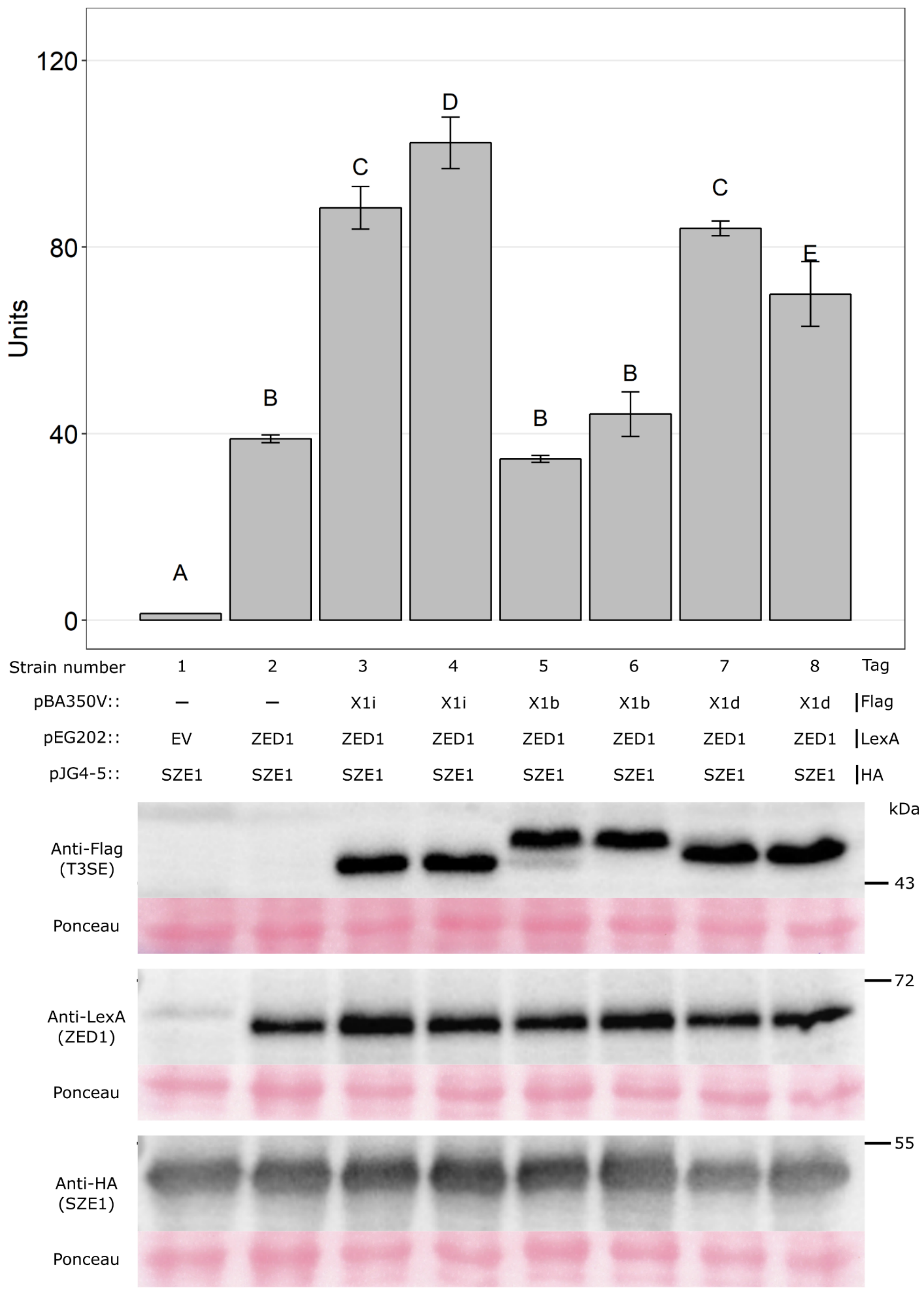

120
80
40
0
Units
D
C
C
E
B
B
B
A
Strain number
1
2
3
4
5
6
7
8
Tag
pBA350V::
—
—
X1i
X1i
X1b
X1b
X1d
X1d
Flag
pEG202::
EV
ZED1
ZED1
ZED1
ZED1
ZED1
ZED1
ZED1
LexA
pJG4-5::
SZE1
SZE1
SZE1
SZE1
SZE1
SZE1
SZE1
SZE1
HA
kDa
Anti-Flag
(T3SE)
43
Ponceau
72
Anti-LexA
(ZED1)
Ponceau
55
Anti-HA
(SZE1)
Ponceau

Supplementary Figure 6 Solely ETI-eliciting HopX1 alleles strengthen ZED1-SZE1 protein-protein interaction. Quantitative yeast interaction assays were performed between ZED1 and SZE1 in the presence or absence of the T3SEs HopX1i (X1i), HopX1b (X1b), or HopX1d (X1d). Letters represent statistically significant differences (Tukey's HSD, $P < 0.05$). Experiments were replicated three times with similar results. Plasmids expressing each component for every strain used are listed below the bar graph. The tag conferred by each vector is listed on the right. Similar protein accumulation levels for all components co-expressed within the same yeast strain was confirmed by western blotting. We tested 2 independently generated strains for all constructs harboring a HopX1 allele. All of these strains harbor pEG202::ZED1 and pJG4-5::SZE1; additionally, strains 3 and 4 harbor pBA350V::HopX1i, strains 5 and 6 harbor pBA350V::HopX1b, and strains 7 and 8 harbor pBA350V::HopX1d.
